# Quantitative Gait Analysis and Cerebrospinal Fluid Tap Test for Idiopathic Normal-pressure Hydrocephalus

**DOI:** 10.1038/s41598-019-52448-3

**Published:** 2019-11-07

**Authors:** Yong-Hyun Lim, Pan-Woo Ko, Ki-Su Park, Sung Kyoo Hwang, Sung-Hee Kim, Jaehwan Han, Uicheul Yoon, Ho-Won Lee, Kyunghun Kang

**Affiliations:** 1 Center of Self-Organizing Software-Platform, Kyungpook National University, Daegu, South Korea; 2 Department of Neurology, School of Medicine, Kyungpook National University, Daegu, South Korea; 3 Department of Neurosurgery, School of Medicine, Kyungpook National University, Daegu, South Korea; 4 Department of Medical and Biological Engineering, Graduate School, Kyungpook National University, Daegu, South Korea; 5 Department of Biomedical Engineering, Daegu Catholic University, Gyeongsan-si, South Korea

**Keywords:** Diagnostic markers, Hydrocephalus, Neurodegenerative diseases

## Abstract

We investigated gait performance utilizing a quantitative gait analysis for 2 groups: (1) idiopathic normal-pressure hydrocephalus (INPH) patients who had a positive response to the cerebrospinal fluid tap test (CSFTT) and (2) healthy controls. The aims of the study were (1) to analyze the characteristics of gait features, (2) to characterize changes in gait parameters before and after the CSFTT, and (3) to determine whether there was any relationship between stride time and stride length variability and Frontal Assessment Battery (FAB) scores in INPH patients. Twenty-three INPH patients and 17 healthy controls were included in this study. Compared with healthy controls, the gait of INPH patients was characterized by lower velocity, shorter stride length, and more broad-based gait. Patients with INPH had a longer stance phase with increased double-limb support. Variability in stride time and stride length was increased in INPH patients. Stride time and stride length variability were correlated with FAB score. After the CSFTT, gait velocity, stride length, and step width significantly improved. There were significant decreases in stride time and stride length variability. These results suggest that the CSFTT for INPH patients might improve the so-called balance-related gait parameter (ie, step width) as well. Stride time and stride length variability also responded to the CSFTT. Association between FAB scores and both stride time and stride length variability suggests involvement of similar circuits producing gait variability and frontal lobe functions in INPH patients.

## Introduction

Idiopathic normal-pressure hydrocephalus (INPH) is an uncommon neurological disorder. Of 563 autopsy cases showing neuropathology of dementia, only 9 (1.6%) cases were suspected as INPH^1^. Despite a low incidence, the diagnosis of INPH is important because INPH is considered a potentially treatable neurological disorder^2^. INPH is an adult-onset syndrome of uncertain origin that involves nonobstructive enlargement of the cerebral ventricles, and is characterized by symptoms of gait disturbance, cognitive impairment, and urinary dysfunction^3^. Although INPH patients present with each of these classic clinical symptoms to varying degrees, the most common and important clinical feature of INPH is gait disturbance^4^.

The cerebrospinal fluid tap test (CSFTT) is considered valuable for predicting shunt effectiveness in INPH patients^5^. The CSFTT has a high positive predictive value for successful shunt surgery^6^. In accordance with the Japanese guideline, clinical improvement after the CSFTT increases diagnostic certainty of INPH from possible to probable^6^.

The GAITRite gait analysis system employs a portable walkway embedded with pressure sensors that detect footfalls as the subject walks the length of the mat^7^. The software enables the documentation of a wide range of temporo-spatial gait parameters, including walking speed, cadence, stride length, step width, and foot placement angles^7^. Its validity and reliability have been proved in various patient populations^8–10^. A few studies on gait changes after the CSFTT have used clinical gait scores rather than quantitative gait analysis procedures^11,12^, resulting in a limited interpretation of their findings. These studies suggest that equilibrium-related symptoms can be improved^11,12^. But, there have been 2 quantitative studies of 10 and 11 INPH patients, respectively, which showed balance-related parameters remained unaffected after the CSFTT^13,14^. However, those were small studies, and further quantitative evidence is needed.

Recent studies have hypothesized an association between INPH and frontal lobe dysfunction. For example, previous reports on INPH brain perfusion patterns have shown diffuse or frontal-focused blood flow reduction in the brain^15,16^. The pathogenesis of gait disturbance in INPH patients is not well understood^11^. However, it has been hypothesized that INPH gait may be caused by frontal dysfunction^17^. Stride time and stride length variability are both parameters that are related to the control of the rhythmic stepping mechanism^18–21^. Impairment in the ability to maintain a steady gait, with minimal stride-to-stride variations, has been known to be closely related to postural instability and fall risk^22^. The Frontal Assessment Battery (FAB) has been known as a short bedside cognitive and behavioral test to assess frontal lobe functions^23^. However, there has been no study to date on any association between FAB and stride time and stride length variability.

In this study, we investigated gait performance utilizing a quantitative gait analysis in 2 groups: (1) INPH patients who had a positive response to the CSFTT and (2) healthy controls. The aims of the study were (1) to analyze the characteristics of gait features, (2) to characterize changes in gait parameters before and after the CSFTT, and (3) to determine whether there was any relationship between stride time and stride length variability and FAB scores in INPH patients.

## Methods

### Participants

Patients in the study were prospectively recruited from patients at the Center for Neurodegenerative Diseases of Kyungpook National University Chilgok Hospital, South Korea between August 2017 to July 2018. All study participants gave informed and written consent for study, including information related to clinical data and MRI. Each patient also consented to having a CSFTT. This study protocol was approved by the Institutional Review Board of Kyungpook National University Chilgok Hospital. All methods and procedures were performed in accordance with relevant guidelines and regulations. INPH diagnosis was made using criteria proposed by Relkin *et al*.^24^. Patients had to be older than 40 years of age with an insidious progression of 6 months or more of INPH symptoms (gait disturbance plus at least 1 other area of impairment in either cognition, urinary symptoms, or both) and have normal CSF opening pressure. Brain MRI showed widening of the ventricles (Evans’ ratio >0.3) for all patients and no macroscopic obstruction of CSF flow. Exclusion criteria included patients with stroke, a recent history of heavy alcohol use, a history of hospitalization for a major psychiatric disorder, or a history of other neurological, metabolic, or neoplastic disorders that might produce dementia symptoms or parkinsonism. No patient in the study showed evidence of head trauma, intracerebral hemorrhage, meningitis, or another known cause of secondary hydrocephalus.

Criteria for healthy control categorization were as follows: no active neurological, systemic, or psychiatric disorders; normal neurological status in examination; and the ability to function independently. Global cognition of healthy controls was assessed by the Korean-Mini Mental State Examination (K-MMSE). Healthy individuals older than 70 years of age also had cranial MRI to exclude any intracranial abnormalities.

### Assessing illness severity

Comprehensive clinical scales for all INPH patients in the study were determined in the following manner. Patients’ dementia severity and general cognition were evaluated with the K-MMSE and Clinical Dementia Rating Scale (CDR)^25,26^. The FAB was used to assess frontal lobe symptoms^23^. Total FAB score ranged from 0 to 18, with a higher score meaning a better performance. The INPHGS was used to assess the severity of each main symptom of INPH (cognitive impairment, gait disturbance, and urinary disturbance) following an unstructured interview with patients and caregivers^27^. The score for each symptom ranges from 0 to 4. Grade 0 indicates normal; grade 1 indicates subjective symptoms but no objective disturbance; grades 2, 3 and 4 indicate mild, moderate, and severe disturbance, respectively. Assessment of gait included timed performance results on the Timed Up and Go (TUG) test and 10 meter walking test^27–30^. The TUG test measures the time it takes a patient sitting in a chair to stand up, walk forward 3 meters, and return to a seated position. Gait disturbance features related to INPH were determined using the Gait Status Scale (GSS)^27^. This scale focuses on 8 factors related to gait disturbance: (1) postural stability; (2) independence in walking; (3) wide base gait; (4) lateral sway; (5) petit-pas gait; (6) festinating gait; (7) gait freezing; and (8) disturbed tandem walking. A total GSS score of the 8 items, ranging from 0 to 16, was determined. A higher score reflected more severe symptoms. Most of these scales were not determined for healthy controls, as selection for healthy controls required a normal neurological examination.

### Cerebrospinal fluid tap test

A lumbar tap removing 30–50 ml of CSF was done for all INPH patients. After the CSFTT, patients were evaluated again with the INPHGS, which is a validated scale for measuring INPH symptom severity, and the TUG test. Gait changes were evaluated 1 day after the CSFTT, and cognition and urination changes were evaluated at 1 week^31^. CSFTT responses were determined with these scales. Responders were identified using the following criteria: greater than 10% improvement in time on the TUG test or improvement of 1 point or more on the INPHGS^6,31^.

### Quantitative gait assessment

A computer-based, 5.8-m-long, pressure-sensitive carpet system (GAITRite, CIR System, Havertown, PA) with a sampling rate of 120 Hz was used to asses gait. Temporal and spatial gait cycle parameters related to this study were recorded. All participants were told to walk barefoot at a comfortable and self-selected speed without the use of any walking aid or a cane. The process was repeated 4 times to obtain sufficient data for analysis, and the mean values obtained from walking 4 times were used in the final analysis. To prevent effects related to acceleration and deceleration, participants started walking 1 m before reaching the active area of electronic walkway and completed their walk 1 m beyond it. Each patient was given time to rest when requested between walking trials to avoid fatigue. Patients always had a researcher walking alongside as a safeguard. Spatiotemporal gait parameters were measured using the GAITRite system as follows: gait velocity, cadence, stride length, step width, toe in/out angle, stride time, stance phase (%), and swing phase (%). The coefficient of variation (CV) for stride time and stride length were calculated as follows: SD of parameter × 100/mean of parameter. Each INPH patient was analyzed twice, once before the CSFTT and once 1 day after the CSFTT.

### Statistical analyses

The IBM SPSS Statistics for Windows version 25.0.0 was used for analyses of data. The demographic data were compared between the INPH and control groups. Fisher’s exact and chi-square tests were used to compare categorical variables, while the Student t tests and Mann-Whitney U tests were used to compare continuous variables. The changes in quantitative gait parameters before and after the CSFTT were analyzed using the repeated-measures analysis of variance. Pearson’s or Spearman’s correlations were employed to investigate the relationship between stride time and stride length variability and FAB scores in INPH patients. Statistical significance was set at *P* < 0.05.

## Results

Table 1 lists the demographic and clinical features for INPH and control subjects. There were no significant differences in the distributions of age and gender between the 2 groups. Patients with INPH had significantly lower K-MMSE scores than the control subjects.

**Table 1:** Demographic data and clinical characteristics of INPH patients and controls at baseline.

Characteristics	INPH (n = 23)	Healthy control (n = 17)	P value
Gender, male	11 (47.8)	4 (23.5)	0.117
Age (year)	73.0 ± 7.0	69.0 ± 5.1	0.052
Education (year)	7.9 ± 4.7	11.3 ± 4.4	0.025
Duration of symptoms (year)	2.4 ± 1.1		
K-MMSE	19.5 ± 5.0	29.2 ± 1.1	<0.001
CDR (0:0.5:1:2:3)	0:10:10:3:0		
**INPHGS**			
GS-Gait	1.0 ± 0.0		
GS-Cogn	2.1 ± 0.5		
GS-Urin	1.9 ± 0.7		
TUG	22.2 ± 25.2		
10-meter walking test	18.4 ± 15.7		
GSS	6.9 ± 3.6		
FAB	9.0 ± 3.3		
Drainage volume of CSF	33.3 ± 3.5		
CSF opening pressure (cm H_2_O)	10.0 ± 3.3		
Evans’ ratio	0.32 ± 0.02		

For INPH patients, data were collected before the CSFTT. Values denote number (%) or mean ± standard deviation. INPH = idiopathic normal-pressure hydrocephalus; CSFTT = cerebrospinal fluid tap test; K-MMSE = Korean version of Mini-Mental State Examination; CDR = Clinical Dementia Rating Scale; INPHGS = Idiopathic Normal-Pressure Hydrocephalus Grading Scale; GS-Gait = INPHGS for gait; GS-Cogn = INPHGS for cognition; GS-Urin = INPHGS for urinary function; TUG = Timed Up-and-Go test; GSS = Gait Status Scale; FAB = Frontal Assessment Battery.

### Differences in gait parameters between patients with INPH and healthy controls

Most gait parameters differed significantly between healthy controls and patients with INPH (Table 2). Compared with healthy controls, the gait of patients with INPH was characterized by a lower velocity (*P* < 0.001) and shorter stride length (*P* < 0.001). INPH patients had significantly higher step width than the control subjects (*P* < 0.001). The toe-out angle was also increased in the INPH group relative to the control group (*P* < 0.01). And, patients with INPH showed a longer stance phase with decreased swing phase than control subjects (*P* < 0.001). Variability in stride time and stride length was increased in INPH patients compared to control subjects (*P* < 0.05 for the CV of stride time and *P* < 0.001 for the CV of stride length).

**Table 2:** Gait parameters in healthy controls and patients with INPH.

	Healthy controls	Patients with INPH	
		Before CSF tap	24 hours after tap
Velocity, cm/s	99.12 ± 10.37	55.12 ± 4.81^c^	67.84 ± 5.01^e^
Cadence, steps/min	110.16 ± 8.81	105.33 ± 3.56	112.99 ± 3.40^d^
Stride length, cm	109.12 ± 4.74	62.76 ± 5.14^c^	72.38 ± 5.11^d^
Step width, cm	7.99 ± 0.75	13.57 ± 0.57^c^	12.90 ± 0.60^d^
Toe in/out, °	8.69 ± 1.61	15.28 ± 1.65^b^	14.57 ± 1.51
Stride time, s	1.09 ± 0.04	1.17 ± 0.04	1.09 ± 0.04^d^
Stance phase, %	62.79 ± 0.69	67.81 ± 0.59^c^	67.28 ± 0.93
Swing phase, %	37.25 ± 0.69	32.19 ± 0.59^c^	32.71 ± 0.93
Double-limb support phase, %	25.61 ± 1.56	36.49 ± 1.34^c^	34.81 ± 1.90
CV of stride time, %	4.34 ± 2.04	10.47 ± 2.27^a^	6.05 ± 0.79^d^
CV of stride length, %	5.10 ± 1.78	14.35 ± 1.98^c^	9.44 ± 0.82^e^

Values denote mean ± standard deviation. INPH = idiopathic normal-pressure hydrocephalus; CSF = cerebrospinal fluid; CV = coefficient of variability.

^a^Mean differs from healthy controls, p < 0.05.

^b^Mean differs from healthy controls, p < 0.01.

^c^Mean differs from healthy controls, p < 0.001.

^d^Mean differs from baseline, p < 0.05.

^e^Mean differs from baseline, p < 0.01.

### Gait parameters in patients with INPH before and after the CSFTT

**Figure 1:**
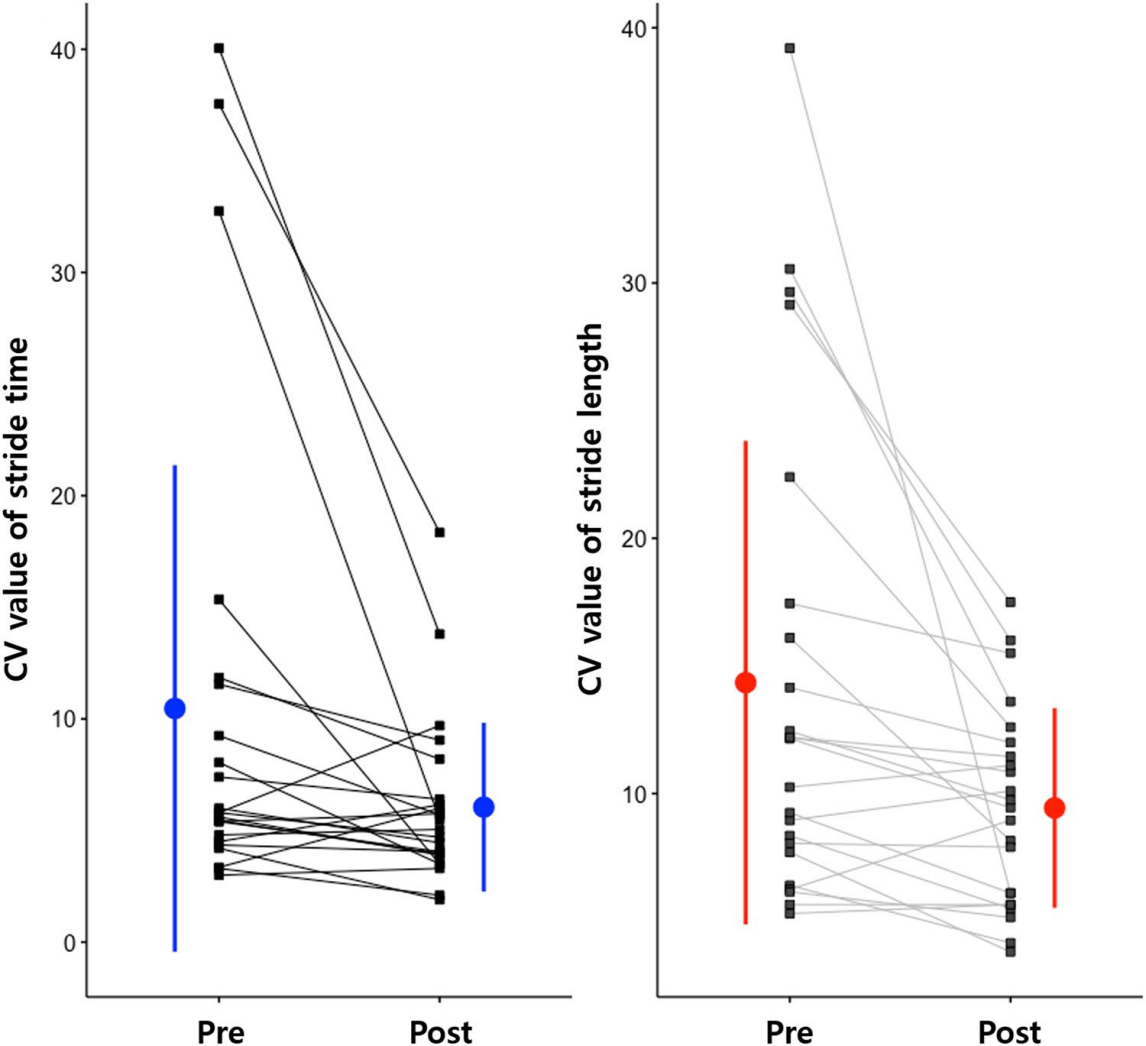
Differences in CV values of stride time and stride length before and after the CSFTT for INPH patients. The mean CV values for each individual before and after the CSFTT are depicted. Filled circles show means and standard deviations.

Differences in the gait parameters before and 24 hours after the CSFTT are shown in Table 2. Gait velocity and stride length improved significantly (*P* < 0.01 for the gait velocity and *P* < 0.05 for the stride length). Step width also improved significantly (*P* < 0.05). Cadence increased significantly (*P* < 0.05). Stride time decreased significantly (*P* < 0.05). Variability in stride time and stride length was improved significantly (*P* < 0.05 for the CV of stride time and *P* < 0.01 for the CV of stride length) (Fig. 1).

### Correlations between FAB Scores and gait variability in INPH

**Figure 2:**
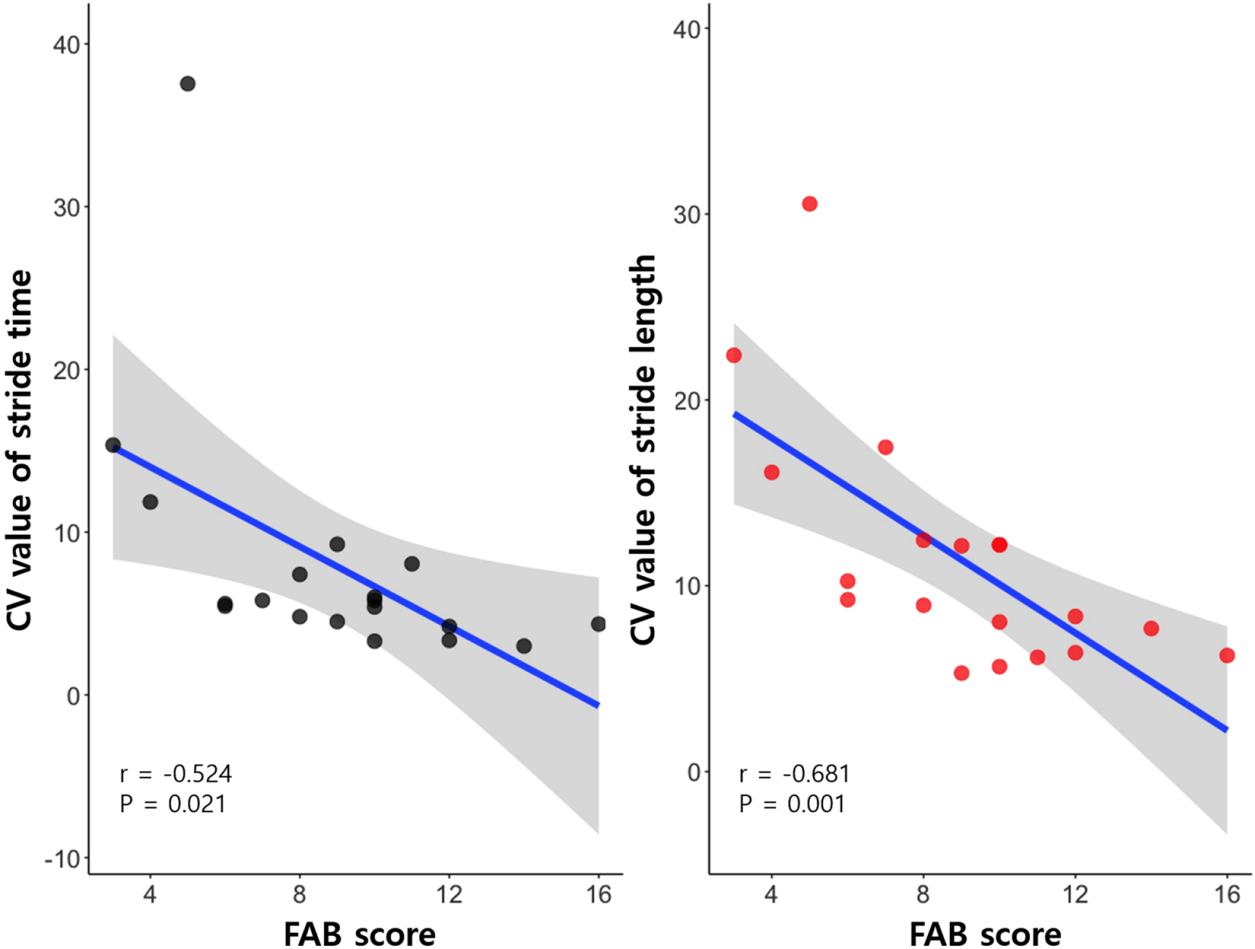
Scatterplots illustrating the relationships between stride time and stride length variability and FAB scores at baseline for INPH patients.

The FAB scores were negatively correlated with the CV value of stride time (r = −0.524; *P* = 0.021) and CV value of stride length (r = −0.681; *P* = 0.001) (Fig. 2).

## Discussion

Compared with age- and gender-matched healthy controls, the gait of patients with INPH was characterized by lower velocity, shorter stride length, and more broad-based gait. Further, patients with INPH had a longer stance phase with increased double-limb support in comparison to control subjects. Gait variability was also significantly higher in the INPH group relative to the control group.

A loss of consistency in the ability to produce a steady gait rhythm, resulting in higher stride-to-stride variability, has been related to balance impairments leading to falls^21,22^. It was reported that increased stride-to-stride variability in stride time and stride length was significantly associated with a high risk for falling in community dwelling older adults^19,32^. Falls are also important clinical problems in patients with INPH^33^. It was reported that more than half of the patients with INPH (56%) experienced falls^33^. We found that stride time and stride length variability were increased in the INPH group. We cautiously suggest that increased gait variability is one of the main risk factors for falls in INPH patients.

Previously unreported, our data showed that stride time and stride length variability were correlated with FAB score. Although the origin of the gait variability in INPH is not totally understood, associations between gait variability measurements and performance on the FAB suggest potentially overlapping processes involved in these functions. Several findings in the literature support these new results for INPH patients. In Alzheimer’s disease, reduction in mean regional cerebral blood flow in the prefrontal cortex was associated with increased stride-to-stride variability^34,35^. Further, some limited evidence in neurodegenerative diseases suggested that the prefrontal cortex was associated with gait variability^35^. Our results bear further connection to previous studies on INPH patients. For example, many previous reports on cerebral perfusion patterns in INPH patients point out diffuse or frontal-dominant reduction in cerebral blood flow^15,16^; in addition, frontal hypoperfusion and frontal subcortical white matter disintegration also have been associated with symptoms in INPH patients including urinary incontinence and gait disturbance^15,36,37^. Moreover, a previous study reported that the total FAB score was correlated with brain single photon emission CT (SPECT) perfusion in the prefrontal cortex independently of age, gender, and MMSE^38^. The study suggested further that the FAB might be useful for evaluating diseases correlated with frontal dysfunction^38^.

Generally, lower body parkinsonism is characteristic in INPH^39^. The aberrant ambulation observed in INPH is characterized by a slow, wide-based gait, and short shuffling steps^40^. Dysfunction of basal ganglia circuitry is known to be mainly responsible for the development of the cardinal features of Parkinson’s disease^41,42^. Ventricular enlargement may interrupt the cortical-subcortical basal ganglia loop, which connects the frontal cortex and basal ganglia, thus resulting in parkinsonism such as bradykinesia and short-stepped gait^43,44^. Considering the connection between cerebral perfusion (also referred to as cerebral blood flow) and brain function^4,45^, and the fact that significant reductions in mean cerebral blood flow of the basal ganglia and the thalamus were found in INPH patients compared with controls^4^, this may also explain the slowed, short-stepped gait observed in our patients.

Both the step width and the foot angle have been generally considered as balance-related gait parameters^13,14^. It is believed that phenomena such as enlarged step width and outward rotated feet can be interpreted as a protective strategy to stabilize gait^13,14^. Cerebellar circuits are well known to be involved in controlling balance^46^. It was also suggested that hydrocephalus may directly compress and therefore impede frontopontocerebellar fibers as they descend close to the lateral ventricle^43^. Considering the fact that a significant reduction in mean cerebral blood flow of the cerebellum was also found for INPH patients compared with controls^4^, our finding showing a broad-based gait pattern with outward rotated feet in INPH patients is not surprising. In our study, patients with INPH may also increase the stance phase and the double-support period to stabilize their inefficient gait control. It has been suggested that both the stance phase and double-limb support are stabilizing factors during normal gait in the elderly^47^.

The CSFTT is usually thought of as an acute treatment for INPH^13^. It has been suggested that CSF movement is not a CSF circulation from the brain ventricles along the entire CSF system to its absorption site in the cortical subarachnoid space, but a permanent rhythmic systolic-diastolic CSF pulsation in all directions along all CSF spaces^48^. CSF production and absorption (CSF exchange) might be constant and present everywhere in the CSF system^49^. Removing 30–50 ml CSF from the lumbar CSF space, as in the CSFTT, may create for a certain period a situation identical to the definitive ventricular shunt operation^5^. Further, the clinical parameters that improve during the CSFTT can be very specific to INPH^13^. Interestingly, our INPH patients showed significant improvements in various gait parameters (especially in the gait velocity, stride length, and step width). At the same time, there were significant decreases in the stride time and stride length variability in our study after CSF removal. Although certain gait parameters, such as the gait velocity and stride length, also improved after the CSFTT in INPH^13^, no previous study has analyzed changes in both stride time and stride length variability after CSF removal. Our results bear further connection to previous studies on INPH patients. For example, the cerebral blood flow in INPH shunt-responders increased postoperatively in the periventricular white matter and the caudate head, a part of the basal ganglia^50^. Additionally, after shunt surgery in INPH patients, local cerebral blood flow increased toward normal, particularly in frontal white matter and basal ganglia^51^. It was suggested that motor function recovery in INPH patients after CSF removal was related to a reversible suppression of frontal periventricular cortico-basal ganglia-thalamo-cortical circuits^52^.

INPH patients were selected consecutively from our prospectively enrolled INPH registry. We tried to minimize any bias related to evaluation before and after the CSFTT by using various objective grading scales. The first limitation of this study is that INPH patients with a negative response to the CSFTT were not included. However, the motivation for this was to enhance diagnostic certainty of INPH by restricting our study to CSFTT responders. In addition, INPH patients that were CSFTT non-responders were generally more likely to have other cerebral comorbidities, which could affect the analysis^53^. Our findings encourage future studies with larger study populations, including both CSFTT responders and non-responders, and quantitative gait parameters to investigate the possibility to utilize a quantitative gait analysis as a neurophysiological biomarker to predict CSFTT response. A second limitation was that we did not investigate quantitative neuroimaging results in our INPH patients. Combining quantitative gait and neuroimaging investigations of INPH patients may help us understand those associations and potentially any underlying pathophysiological interrelationships. Third, this study included a relatively small number of participants. Because of the limited sample size, the results of this study need to be replicated in future studies. To our knowledge, there have been only 2 studies investigating changes in quantitative gait parameters before and after the CSFTT in INPH patients, but these previous studies included only 10 or 11 INPH participants^13,14^.

In conclusion, this study demonstrated that the CSFTT for INPH might improve the so-called balance-related gait parameter (ie, step width). Stride time and stride length variability also responded to the CSFTT. Our findings suggest future studies are needed to investigate whether CSF removal in INPH patients decreases a risk of falling. Further, association between FAB scores and both stride time and stride length variability suggests frontal lobe functions and gait variability in INPH patients may involve similar circuits.

## Data Availability

The datasets generated and analyzed during the current study are available from the corresponding author upon request.
